# A case of long-term survival following hepatectomy for liver metastasis of Merkel cell carcinoma

**DOI:** 10.1186/s40792-015-0015-7

**Published:** 2015-03-18

**Authors:** Katsutoshi Shoda, Hisashi Ikoma, Yusuke Yamamoto, Osamu Kinoshita, Ryo Morimura, Hirotaka Konishi, Yasutoshi Murayama, Shuhei Komatsu, Atsushi Shiozaki, Yoshiaki Kuriu, Masayoshi Nakanishi, Daisuke Ichikawa, Hitoshi Fujiwara, Kazuma Okamoto, Chohei Sakakura, Toshiya Ochiai, Hiroki Taniguchi, Satoshi Yasukawa, Eigo Otsuji

**Affiliations:** Department of Digestive Surgery, Kyoto Prefectural University of Medicine, 465 Kajii-cho, Kamigyo-ku, Kyoto, Japan; Department of Pathology, Kyoto Prefectural University of Medicine, Kyoto, Japan; Department of Surgery, North Medical Center Kyoto Prefectural University of Medicine, Kyoto, Japan; Department of Surgery, Japanese Red Cross Kyoto Daini Hospital, Kyoto, Japan

**Keywords:** Liver metastasis, Merkel cell carcinoma, Hepatectomy

## Abstract

**Background:**

Merkel cell carcinoma (MCC) is a rare endocrine tumor that presents as a rapidly growing skin nodule of the body, and it is aggressive with regional nodal and distant metastases without clearly defined treatment. There are no reports of long survivors among patients with liver metastasis of MCC. The current case was a patient who underwent surgical resection for liver metastasis of Merkel cell carcinoma.

**Case presentation:**

This case describes a 73-year-old female with a papule on the dorsal side of the right third finger and liver tumor. The papule of the right third finger was histologically diagnosed as MCC by the skin biopsy. She underwent extensive resection and lymph node dissection of the right third finger and partial resection of the liver. The liver tumor was histologically diagnosed as liver metastasis of MCC. The patient remains well without any evidence of tumor recurrence more than 5 years after surgery.

**Conclusions:**

This is the first report of a long-term survival of more than 5 years following liver metastasis of MCC, which was surgically resected. Patients with small and solitary metastatic liver tumor of MCC may have a chance for a long-term survival following the hepatic resection.

## Background

Merkel cell carcinoma (MCC) is a relatively rare neuroendocrine cancer in the skin with an age-adjusted incidence of less than 0.5 per 100,000 person-years [1]. Patients with MCC tend to have a poor prognosis with a 5-year survival rate of approximately 50% even after curative tumor resection, because of the high frequency of early metastasis to the lymph nodes, lung, skin, and central nervous system [2]. Clinically localized MCC is treated with wide surgical excision of the primary tumor [3]; however, the therapeutic strategy against distant metastasis of MCC has not yet been standardized. Patients with liver metastasis of MCC rarely have a chance undergoing hepatectomy, and there are no reports of any long-term survivors of liver metastasis of MCC after hepatectomy in the English literature. The current case was a patient who underwent surgical resection for liver metastasis of MCC, and this patient has survived without recurrence for more than 5 years after hepatectomy.

## Case presentation

### Clinical summary

A 73-year-old female was found to have a papule, measuring 20 mm in diameter, on the dorsal side of the right third finger. Laboratory data indicated that her alanine aminotransferase, carcinoembryonic antigen, carbohydrate antigen 19-9, squamous cell carcinoma antigen, neuron specific enolase, and alpha-fetoprotein were within normal limits, but the serum levels of protein induced by vitamin K absence or antagonist II were elevated to 51 mAU/ml. The serum markers for hepatitis B and hepatitis C were negative.

**Figure 1:**
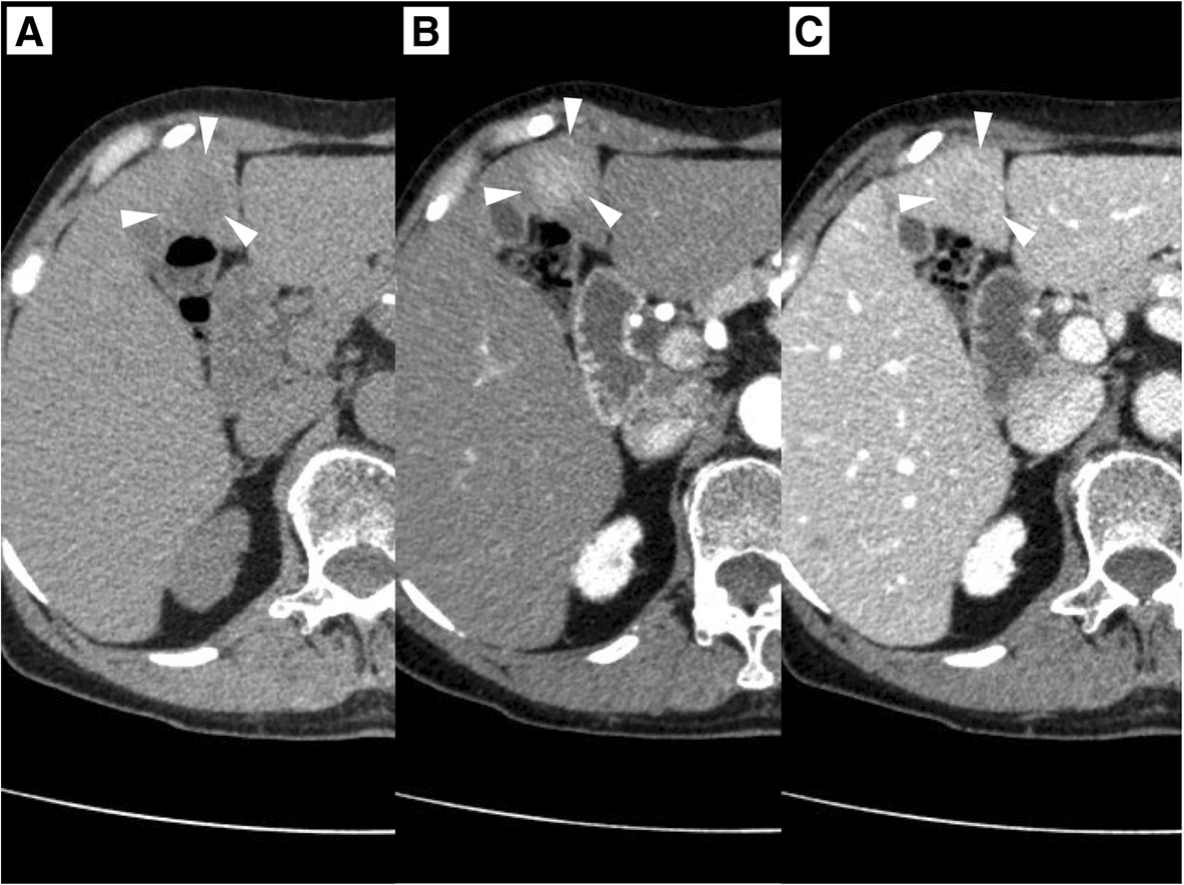
**Preoperative abdominal computed tomography.** Preoperative abdominal computed tomography revealed an ill-defined mass (white arrowhead), 20 mm in diameter, on the liver segment IV **(A)**, which was well-stained in the arterial phase **(B)**, and ring-shaped enhanced in the portal phase **(C)**.

**Figure 2:**
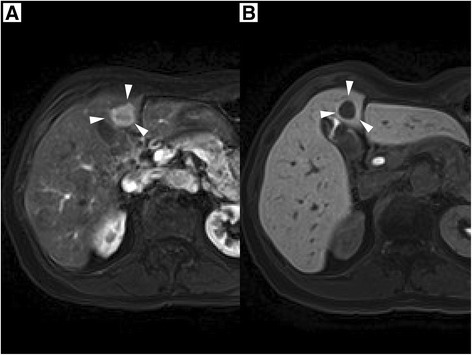
**Gadoxetate disodium-enhanced magnetic resonance imaging.** Gadoxetate disodium-enhanced magnetic resonance imaging showed a well-stained mass (white arrowhead) in the arterial phase **(A)** and a well-defined hypointense mass in the hepatocyte-specific phase **(B)**.

**Figure 3:**
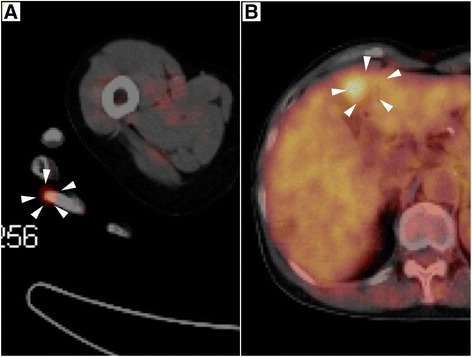
**Positron emission tomography/computed tomographic scans.** Positron emission tomography/computed tomographic scans showed the uptake of 2-[fluorine-18] fluoro-2-deoxy-D-glucose in the right third finger (white arrowhead, **A**) and liver segment IV (white arrowhead, **B**).

Abdominal computed tomography (CT) revealed an ill-defined mass, measuring 20 mm in diameter on liver segment IV, which was well-stained in the arterial phase and ring-shaped enhanced in the portal phase (Figure 1). The tumor appeared as a well-defined hypointense mass in the hepatocyte-specific phase on gadoxetate disodium-enhanced magnetic resonance imaging (Figure 2). Positron emission tomography/computed tomographic scans showed the uptake of 2-[fluorine-18] fluoro-2-deoxy-D-glucose in liver segment IV and the right third finger (Figure 3). She underwent a skin biopsy of the papule on the right third finger in a local hospital, and it was histologically diagnosed as MCC. MCC of the right third finger and metastatic tumor of the liver were suspected. She underwent extensive resection and lymph node dissection of the right third finger at a local hospital. All resection margins were negative and there was no lymph node metastasis.

She was referred to our hospital, and partial resection of liver segment IV was performed under a preoperative diagnosis of the metastatic liver tumor of the MCC. The patient started adjuvant chemotherapy 6 weeks after hepatectomy, and she has received cisplatin (110 mg/body) and etoposide (145 mg/body) for 12 weeks. The patient remains well without any evidence of tumor recurrence more than 5 years after surgery.

### Pathological findings

A histopathological examination of the specimen from the right third finger showed cells with scanty cytoplasm and small round nuclei, proliferating in the trabecular and clumps with fibrosis. The nuclei were clear and pale with delicate chromatin. The tumor was immunohistochemically positive for neuroendocrine markers (synaptophysin and CD56), cytokeratin marker (CAM5.2), and CK20. These findings suggested that pathological diagnosis was MCC on the right third finger.

**Figure 4:**
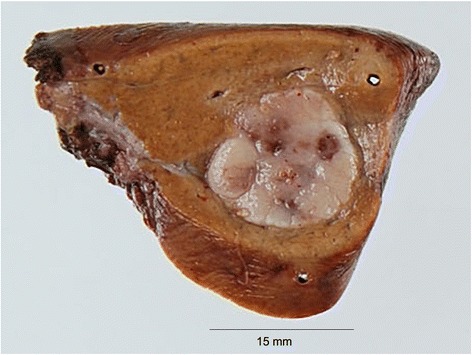
**Gross view of liver tumor.** Gross view of liver tumor shows a yellowish-white, elastic-hard solid component and clear boundary line between the surrounding liver tissue and tumor.

**Figure 5:**
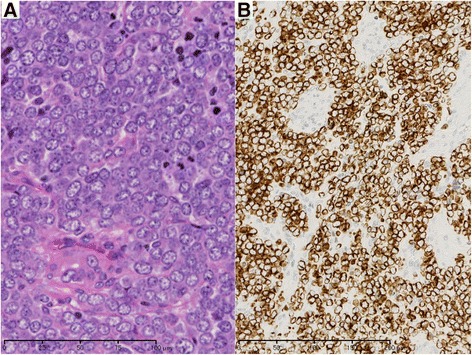
**Histological appearance of high-magnification view of the liver tumor.** Histological appearance of high-magnification view of the liver tumor (**A**, hematoxylin and eosin stain × 400) and immunohistochemical expression (×200) of CK20 (B). **(A)** Cells with scanty cytoplasm and small round nuclei, proliferating in the trabecular and clumps with fibrosis. Nuclei are clear pale with delicate chromatin. **(B)** CK20 is positive. The edge of the tumor was observed in the staining of perinuclar dot pattern, which is typical finding of MCC.

**Figure 6:**
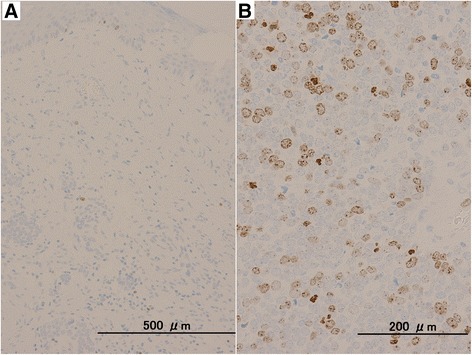
**Representative findings of Ki-67 staining with primary tumor and metastatic liver tumor.** Ki-67 immunohistochemical findings in primary MCC **(A)** and metastatic liver tumor **(B)**. Ki-67 LI of primary MCC was 2.1% and that of liver metastasis was 26.0%.

The resected liver tumor measured 15 × 13 mm (Figure4). The cut surface of the tumor showed a yellowish-white, elastic-hard solid component and clear boundary line between the surrounding liver tissue and tumor. There were cells with scanty cytoplasm and small round nuclei, proliferating in a trabecular pattern and clumps with fibrosis. The nuclei were clear pale with delicate chromatin. The edge of the tumor showed a perinuclear dot-staining pattern, which is typical finding of MCC. The tumor component was similar to the primary tumor of MCC tumor of the right third finger. Neuroendocrine markers (synaptophysin, chromogranin A, and CD56), cytokeratin marker (CAM5.2), neurofilament, and CK20 were immunohistochemicaly positive, and S100 was negative. Figure 5A, B shows representative findings of hematoxylin-eosin staining and CK20 immunohistochemistry with metastatic liver tumor, respectively. These findings suggested that the pathological diagnosis was liver metastasis of MCC. The tumor was not exposed on either the surface or the cut surface of the liver. Additional pathological results revealed that primary tumor showed a low proliferative labeling index (Ki-67: 2.1%), and metastatic liver tumor showed a high proliferative index (Ki-67: 26.0%). Figure 6A, B shows representative findings of Ki-67 staining with primary tumor and metastatic liver tumor, respectively.

## Discussion

MCC is a rare, aggressive neuroendocrine tumor that is usually found in the dermis and can extend into the subcutaneous tissue or involve the overlying epidermis [4]. Patients with distant metastasis have a poor prognosis due to the high propensity for local recurrence and distant metastasis [5]. Median overall survival with MCC was 3.6 years for local disease, 2.0 years for regional disease, and 1.2 years for distant metastasis in multicenter cohort study [6]. This is the first case of long survival of more than 5 years after hepatectomy for liver metastasis of MCC without recurrence.

Gastroenteropancreatic neuroendocrine tumor is generally classified by mitotic count and Ki-67 labeling index (Ki-67 LI), according to the WHO 2010 classification. Some researcher recently suggested that the higher Ki-67 LI might be associated with unfavorable oncological outcome [7]. In this study, the Ki-67 LI of primary MCC was 2.1% and that of liver metastasis was 26.0%. This result suggests two important implications. First, primary MCC with lower Ki-67 LI can develop metastasis. Second, the complete resection for liver metastasis may contribute to the long survival, even if the tumor has high Ki-67 LI.

MCC sometimes develops metastasis to the lymph nodes, kidney, small bowel, pancreatic body, adrenal glands, abdominal wall, bone marrow, and parathyroid glands [2]. The frequency of liver metastases is relatively rare [8], and patients with liver metastasis of MCC rarely have a chance undergoing hepatectomy. Skagias et al. reported a case that underwent extended right hepatectomy for the liver metastasis of MCC, 15 cm in diameter, which is the only report of hepatic resection for liver metastasis of MCC [9]. They provided no data of the prognosis after hepatectomy. The current metastatic tumor of the liver was solitary, and the maximum diameter of the liver tumor was 15 mm. It is difficult to make any definite comments; however, the current results suggest that patients with small and solitary metastatic liver tumor of MCC may achieve long-term survival following hepatic resection, and hepatic resection may be justified for these patients. Thus, careful examination of the liver is important after primary resection of MCC, and early detection of the liver metastasis of MCC is crucial.

The typical chemotherapeutic regimens of MCC are similar to those for small cell lung cancer and neuroendocrine tumors in other locations [1,10]. The optimal chemotherapy regimen for patients with MCC remains controversial, although most authors have prescribed combination chemotherapy regimens [8]. Some common regimens include cisplatin with etoposide, cytoxan with adriamycin and vincristine, cisplatin with adriamycin, and streptozocin with 5- fluorouracil [11,12]. The reported median survival time from initiation of chemotherapy for distant metastasis of MCC is approximately 9 months [5,13]. The current patient received chemotherapy with cisplatin and etoposide for 12 weeks, beginning 6 weeks after hepatectomy for liver metastasis of MCC. Although the current patient received chemotherapy according to the previous report [11], MCC was initially thought to be resistant to chemotherapy. In recent study, it was suggested that treating with adjuvant chemotherapy was not associated with improved MCC outcomes in large cohort [6]. On the other hand, in some previous reports, adjuvant radiation therapy plays a significant role in MCC treatment by reducing recurrence and improving local/regional control of disease [3,14,15]. Moreover, some authors mentioned that neoadjuvant chemotherapy led to local control and allowed curative surgery [16], resulting in long-term survival, whereas the clinical results of these therapies cannot be verified without long survivors. We think that a curative surgery with enough margins is the best choice contributing to improvement of the outcome, and further prospective study with neoadjuvant and adjuvant therapy will be needed.

## Conclusions

In conclusion, this is the first report of a long-term survival of more than 5 years following liver metastasis of MCC, which was surgically resected. Patients with small and solitary metastatic liver tumor of MCC may have a chance for a long-term survival following the hepatic resection.

## Consent

Written informed consent was obtained from the patient for publication of this case report and any accompanying images. A copy of the written consent is available for review by the Editor-in-Chief of this journal.

## Abbreviation

MCC: Merkel cell carcinoma
